# Cloning of a Passage-Free SARS-CoV-2 Genome and Mutagenesis Using Red Recombination

**DOI:** 10.3390/ijms221910188

**Published:** 2021-09-22

**Authors:** Alexandra Herrmann, Doris Jungnickl, Arne Cordsmeier, Antonia Sophia Peter, Klaus Überla, Armin Ensser

**Affiliations:** Institute for Clinical and Molecular Virology, Friedrich-Alexander University Erlangen-Nürnberg (FAU), 91054 Erlangen, Germany; alexandra.herrmann@uk-erlangen.de (A.H.); doris.jungnickl@uk-erlangen.de (D.J.); arne.cordsmeier@uk-erlangen.de (A.C.); antoniasophia.peter@uk-erlangen.de (A.S.P.); klaus.ueberla@fau.de (K.Ü.)

**Keywords:** SARS-CoV-2, COVID-19 pandemic, reverse genetics, recombinant virus, bacterial artificial chromosome, red recombination, reporter virus, incoming viral particles

## Abstract

The ongoing pandemic coronavirus (CoV) disease 2019 (COVID-19) by severe acute respiratory syndrome CoV-2 (SARS-CoV-2) has already caused substantial morbidity, mortality, and economic devastation. Reverse genetic approaches to generate recombinant viruses are a powerful tool to characterize and understand newly emerging viruses. To contribute to the global efforts for countermeasures to control the spread of SARS-CoV-2, we developed a passage-free SARS-CoV-2 clone based on a bacterial artificial chromosome (BAC). Moreover, using a Lambda-based Red recombination, we successfully generated different reporter and marker viruses, which replicated similar to a clinical isolate in a cell culture. Moreover, we designed a full-length reporter virus encoding an additional artificial open reading frame with wild-type-like replication features. The virus-encoded reporters were successfully applied to ease antiviral testing in cell culture models. Furthermore, we designed a new marker virus encoding 3xFLAG-tagged nucleocapsid that allows the detection of incoming viral particles and, in combination with bio-orthogonal labeling for the visualization of viral RNA synthesis via click chemistry, the spatiotemporal tracking of viral replication on the single-cell level. In summary, by applying BAC-based Red recombination, we developed a powerful, reliable, and convenient platform that will facilitate studies answering numerous questions concerning the biology of SARS-CoV-2.

## 1. Introduction

Coronaviruses (CoVs) are large, enveloped, single-stranded positive-sense RNA viruses that cause severe disease in both humans and animals. Until 2019, six α- and β-CoVs were known to cause respiratory diseases of varying severities in humans, including viruses that cause symptoms of the common cold (229E, NL63, OC43, and HKU1) and two highly pathogenic CoVs (SARS-CoV and MERS-CoV). In December 2019, a new highly pathogenic and transmissible CoV was isolated from patients with respiratory disease [1]. Since then, the severe acute respiratory syndrome (SARS) coronavirus 2 (CoV-2), the causative agent of the CoV disease 19 (COVID-19), was responsible for over 200,000,000 cases and more than 4,000,000 deaths worldwide [2]. Although potent vaccines are now available, specific antiviral drugs for the prevention and treatment of COVID-19 are urgently needed.

Reverse genetic approaches to generate recombinant viruses are a powerful tool to uncover the biology of viral infections. Infectious molecular clones are highly valuable research tools to better understand the mechanisms of viral infections and to answer important questions regarding virus transmission and pathogenesis. Moreover, recombinant viruses expressing reporter genes facilitate cell culture-based screening assays or small animal models to identify prophylactic or therapeutic compounds. Finally, precise and uncomplicated approaches to manipulate viral genomes are prerequisites to generate live-attenuated vaccine candidates and to identify viral and host factors that are involved in virus biology and immunity.

Several different methods were already used to generate recombinant SARS-CoV-2 viruses, including in vitro ligation [3], homologous recombination in yeast [4], and circular polymerase extension reaction [5,6], as well as plasmid- and bacterial artificial chromosome (BAC)-based approaches [7,8,9].

In this study, we describe the assembly of a passage-free SARS-CoV-2 B.1 genome into a single BAC and its manipulation by Lambda Red-based recombination, which was originally established for the manipulation of herpesvirus genomes [10,11]. We demonstrate that our reverse genetics system can be easily used to recover wild-type and mutant viruses from transfected cells. Red recombination alone or in combination with Gibson assembly were successfully applied to generate different fluorescence and luciferase reporter viruses. Recovered viruses display growth kinetics comparable to the recombinant wild-type (WT) and the clinical isolate. The results obtained within this study demonstrate that our recombinant reporter-expressing SARS-CoV-2 variants represent a reliable tool for the identification of new therapeutics for the prevention and treatment of COVID-19, as shown for the two reference drugs remdesivir (RDV) and GC376. Notably, we designed a marker virus that enables the visualization of incoming viral particles on a single-cell level. Collectively, our methodological platform represents an ideal tool for the fast and easy cloning of modified viruses to promote drug development, live-attenuated vaccine candidates, and a better understanding of the transmission and pathology of constantly emerging variants of concern.

## 2. Results

### 2.1. Assembly of pBelo-SARS-CoV-2 (pBSCoV2)

**Figure 1:**
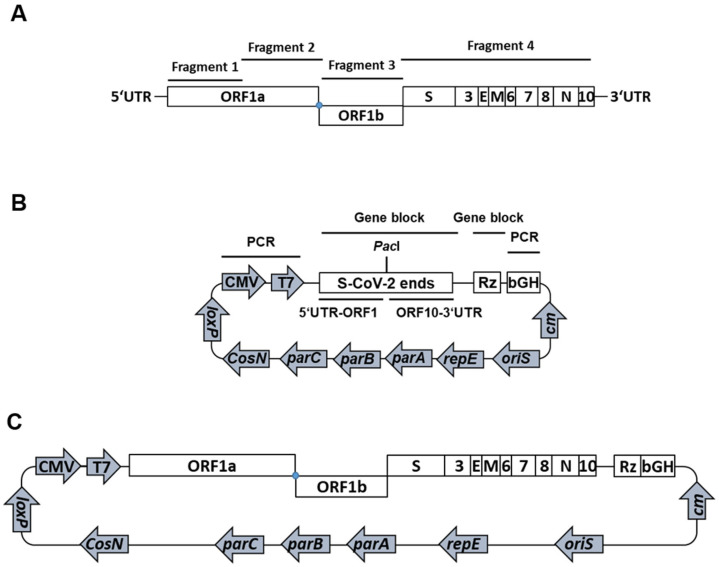
Cloning of a passage-free SARS-CoV-2 genome into a bacterial artificial chromosome (BAC). (**A**) Amplification of four overlapping fragments covering the entire SARS-CoV-2 genome. The open reading frames (ORFs) and structural and accessory proteins are indicated. Extracted viral nucleic acids of a respiratory swab sample served as the template. The genome structure is not to scale. (**B**) Schematic depiction of the modified pBeloBAC11 backbone pBeloCoV. Human cytomegalovirus (CMV) and T7 RNA polymerase (T7) promoters, as well as the bovine growth hormone polyA tail (bGH), were amplified from pcDNA4 and cloned into pBeloBAC11 together with gene blocks encoding the 5′ and 3′ ends of the SARS-CoV-2 genome with a flanking *Pac*I site and the hepatitis delta virus (HDV) ribozyme (Rz). (**C**) Schematic illustration of pBelo-SARS-CoV-2 (pBSCoV2). The four amplified fragments were assembled with the *Pac*I-linearized pBeloCoV backbone.

For the cloning of a passage-free SARS-CoV-2 genome into a single bacterial artificial chromosome (BAC), we used viral RNA from an anonymized respiratory swab sample of a patient with SARS-CoV-2 infection (GISAID EPI_ISL_2732373). Extracted nucleic acids of the patient sample served as a template for cDNA synthesis and the subsequent amplification of four PCR fragments covering the entire viral genome (Figure 1A). The resulting fragments were assembled with a modified, linearized pBeloBAC11 backbone termed pBeloCoV (Figure 1B).

We inserted both CMV and T7 promoters, the 5′ and the 3′ ends of the SARS-CoV-2 genome separated by a *Pac*I endonuclease cleavage site, the hepatitis virus D (HDV) ribozyme, and a bGH termination and polyadenylation signal into the pBeloBAC11 vector (Figure 1B). The CMV promoter allows the transcription of the viral genome upon transfection into mammalian cells, whereas the T7 promoter provides an option for in vitro transcription and may also be used for a more efficient transcription in cells stably expressing the T7 RNA polymerase. The ribozyme and the polyadenylation signal are required to create a genomic RNA with an authentic 3′ end. The initial transformation of assembled BACs only yielded incomplete clones, lacking most of fragment 2, as demonstrated by restriction digestion and next-generation sequencing (NGS).

To obtain clones with full-length genomes, we applied the standard Lambda Red recombination [10,11] to insert fragment 2 (Figure 1A) into an otherwise correctly assembled clone (Figure S1). First, a kanamycin resistance cassette with short-sequence duplications complementary to the SARS-CoV-2 genome at the site of recombination and flanking single-cutting restriction sites was amplified. Upon electroporation of the kanamycin cassette into *E. coli* GS1783 containing the incomplete SARS-CoV-2 clone and the initiation of recombination, kanamycin-resistant clones were screened by colony PCR and restriction digestion. We removed the kanamycin cassette from two positive clones by endonuclease digestion and used the resulting linear DNA for assembly with the lacking fragment 2. The resulting pBelo-SARS-CoV-2 (pBSCoV2) clones containing the full-length SARS-CoV-2 genome were confirmed by restriction digestion and NGS. The sequence analysis identified five mutations compared to the original Wuhan sequences that were already present in the clinical specimen used for cDNA synthesis: 5′UTR-T241C, Nsp2-T265I, Nsp12-P323L, Spike-D614G, and ORF3a-Q57H (B.1 by Pangolin nomenclature; in GISAID hCoV-19/Germany/pBSCoV2-K49/2020, EPI_ISL_2732373).

### 2.2. Recovery of Recombinant (Rec) SARS-CoV-2

**Figure 2:**
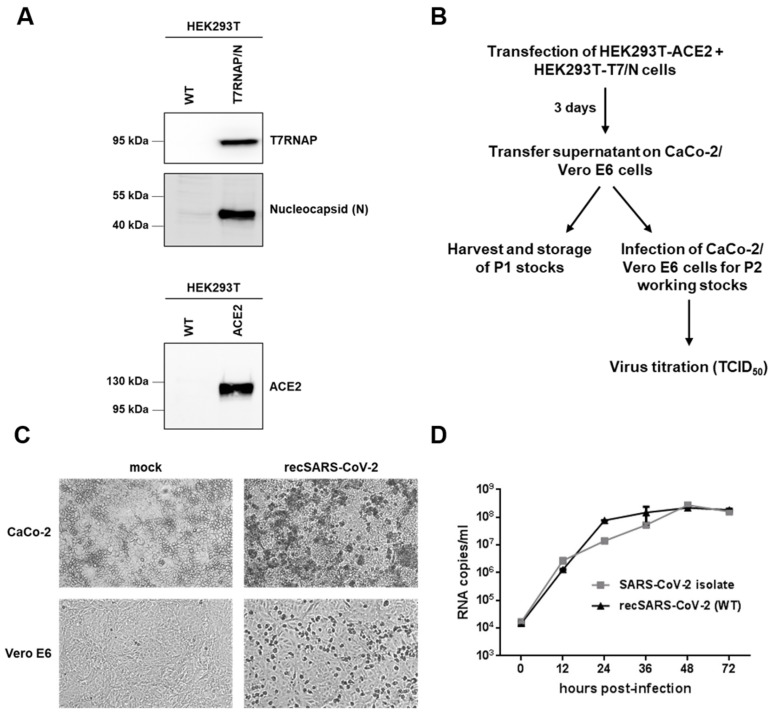
Rescue of recSARS-CoV-2. (**A**) Western blot analysis of HEK293T cells stably expressing either T7 RNA polymerase (T7RNAP) and the viral nucleocapsid protein (N) or the ACE2 receptor upon lentiviral transduction. (**B**) Illustration of the approach followed to rescue recSARS-CoV-2. A coculture of HEK293T-ACE2 and HEK293T-T7RNAP/N cells was transfected with pBSCoV2. After three days, the supernatant was transferred on CaCo-2 or Vero E6 cells to obtain the first passage (P1) virus stocks. P1 stocks were used to infect CaCo-2 and Vero E6 cells for the P2 working stocks, respectively. The virus titers were determined by TCID_50_ titration. (**C**) Visualization of the cytopathic effect (CPE) in recSARS-CoV-2 infected CaCo-2 and Vero E6 cells three days after infection. Mock-infected cells are shown as the control. (**D**) Growth kinetics of a SARS-CoV-2 clinical isolate compared to recSARS-CoV-2. CaCo-2 cells were infected with the clinical isolate or the recombinant SARS-CoV-2 virus at an MOI of 0.005. Cell culture supernatants were collected at the indicated time points post-infection, and viral copies were determined by RT-qPCR targeting the viral polymerase RdRp. Data are presented as the means of triplicates ± SD.

To reconstitute recombinant SARS-CoV-2, we transfected the pBSCoV2 BAC into a coculture of HEK293T cells stably expressing either the ACE2 receptor or the viral N protein and T7 RNA polymerase (Figure 2A). Three days post-transfection, we transferred the supernatant on CaCo-2 or Vero E6 cells (Figure 2B). The cells were monitored daily for the presence of the cytopathic effect (CPE), which was evident in both cell lines after three days (Figure 2C).

For the subsequent analyses, the supernatants were further passaged on CaCo-2 or Vero E6 cells to obtain high titer P2 working stocks (Figure 2B). In parallel, virus stocks for the clinical isolate (MUC-IMB-1; also B.1. by Pangolin nomenclature) were prepared on the same cell type. The amount of infectious particles for both strains was determined by endpoint titration (TCID_50_). To confirm that the replication of our reconstituted recombinant SARS-CoV-2 clone is comparable to this clinical isolate, we performed replication kinetics in the CaCo-2 cells and quantified the genomic RNA copies by RT-qPCR (Figure 2D). Although slightly more genomic RNA copies were detected in the supernatant of the cells infected with recSARS-CoV-2 after 24 and 36 h post-infection (hpi), the virus showed a very similar growth rate and peak viral titers comparable to the clinical isolate. Thus, both findings demonstrated that we successfully reconstituted a recombinant SARS-CoV-2 clone, which exhibited replication characteristics comparable to the clinical isolate.

### 2.3. Generation and Characterization of recSARS-CoV-2 Reporter and Marker Viruses

**Figure 3:**
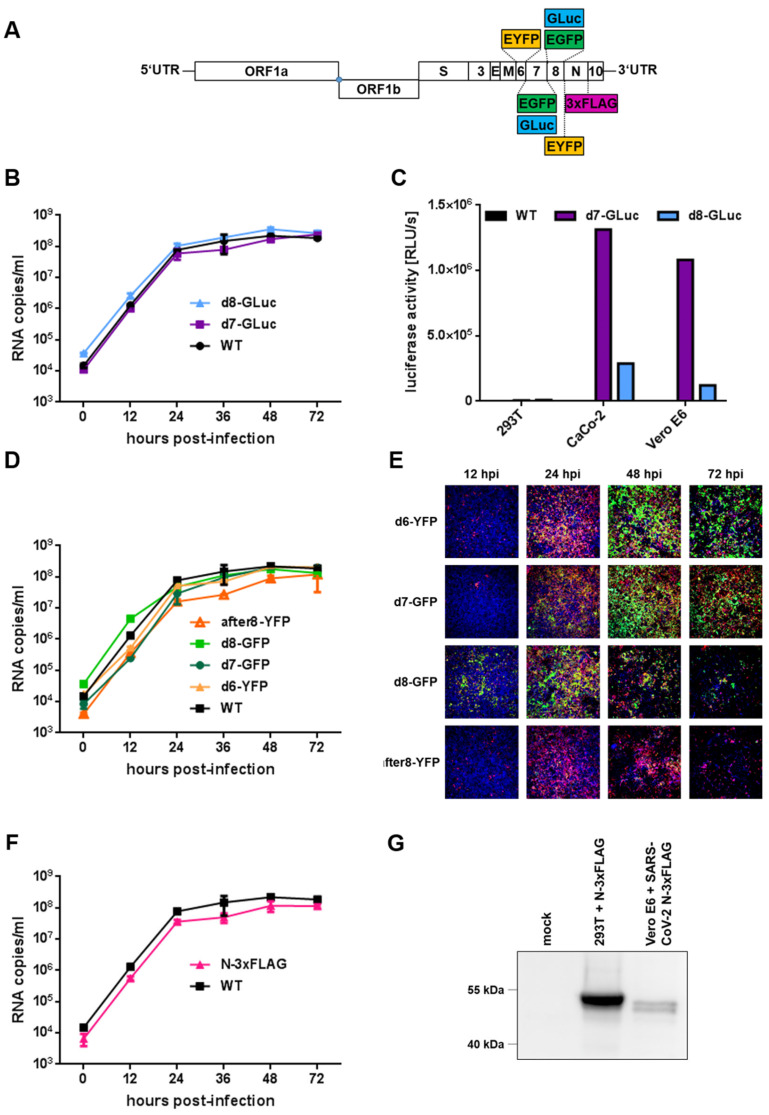
Generation and characterization of recSARS-CoV-2 reporter viruses. (**A**) Schematic depiction of recSARS-CoV-2 reporter and marker viruses. The illustration is not to scale. (**B**) Replication kinetics of luciferase reporter viruses. CaCo-2 cells were infected with recSARS-CoV-2 WT, as well as reporter viruses expressing gaussia luciferase (GLuc) instead of viral ORF7a (d7) and ORF8 (d8), respectively (MOI = 0.005). Cell culture supernatants were collected at the indicated time points post-infection, and viral RNA was quantified by RT-qPCR targeting the viral polymerase RdRp. (**C**) Luciferase assay for the detection of viral replication. HEK293T cells were transfected with pBSCoV2 (WT), pBSCoV2-d7-GLuc, or pBSCoV2-d8-GLuc. After 3 days, the supernatant was passaged on to CaCo-2 or Vero E6 cells. Cell culture supernatants were analyzed for GLuc activity three days post-transfection and post-infection, respectively. Luciferase activity is depicted as relative light units per second (RLU/s). (**D**) Replication kinetics of fluorescence reporter viruses. CaCo-2 cells were infected at an MOI of 0.005 with recSARS-CoV-2 WT and different reporter viruses expressing either GFP or YFP instead of viral ORF6 (d6), ORF7a (d7), and ORF8 (d8), or as an additional gene between ORF8 and N (after8). Cell culture supernatants were collected at the indicated time points, and viral RNA was quantified by RT-qPCR. (**E**) Fluorescence reporter expression. CaCo-2 cells were infected with the indicated fluorescence reporter viruses (MOI = 0.005). Cells were fixed at 12, 24, 48, and 72 hpi; permeabilized; immunostained with mAb against the spike protein (red); and visualized for reporter expression (green). Hoechst 33342 was used for nuclear staining (blue). Imaging was performed with an ImmunoSpot Image Analyzer, and representative overlays of the respective signals are depicted. (**F**) Replication kinetics of the N-3xFLAG marker virus. CaCo-2 cells were infected with recSARS-CoV-2 WT and a marker virus that expresses 3xFLAG-tagged N (MOI = 0.005). Cell culture supernatants were collected at the indicated time points post-infection, and viral RNA was quantified by RT-qPCR. (**G**) Western blot analysis of N-3xFLAG. Cell lysates of Vero E6 cells infected with the N-3xFLAG marker virus were analyzed for the expression of 3xFLAG-tagged nucleoprotein using an antibody specifically recognizing the tag sequence. Uninfected (mock) and HEK293T cells transfected with a N-3xFLAG expression vector served as the negative and positive controls, respectively.

Next, the Lambda-based Red recombination system and Gibson assembly were used to generate recombinant viruses that express fluorescence or luciferase reporter genes. We replaced open reading frame (ORF) 7a or ORF8 with either EGFP or gaussia luciferase (GLuc) and ORF6 with EYFP, resulting in pBSCoV2-deltaORF7-GFP (d7-GFP), -deltaORF7-GLuc (d7-GLuc), -deltaORF8-GFP (d8-GFP), -deltaORF8-GLuc (d8-GLuc), and -deltaORF6-YFP (d6-YFP), respectively (Figure 3A). Moreover, we cloned a full-length reporter virus that encodes EYFP as an additional artificial ORF between ORF8 and N (after8-YFP). Upon infection, EYFP is expressed from a subgenomic RNA due to the insertion of an additional TRS sequence (Figure 3A). The integrity of the newly obtained BACs was confirmed by a restriction analysis and NGS, and reporter viruses were recovered according to the previously established procedure (Figure 2B). To assess the possible effects of the replacement of viral ORFs with gaussia luciferase on viral fitness, we first compared the growth kinetics of d7-GLuc and d8-GLuc with the recombinant WT (Figure 3B). Quantitative RT-qPCR revealed similar growth kinetics and peak viral titers for both luciferase reporter viruses in comparison to the WT, suggesting that the replacement of either ORF by GLuc did not significantly affect the viral fitness in vitro. Next, we evaluated reporter expression by the quantification of GLuc activity in cell culture supernatants (Figure 3C). We used supernatants of either pBSCoV2-transfected HEK293T cells (3 days post-transfection) or CaCo-2 and Vero E6 cells 3 days after passaging of the 293T supernatant (P1 viruses; Figure 2B), respectively. Recombinant WT was used as a negative control. Interestingly, we could not detect luciferase activity in the transfected HEK293T cells, although they produce infectious viruses, which might be the result of the low transfection rates. However, upon passaging on the CaCo-2 and Vero E6 cells, a reporter signal for both the d7- and d8-GLuc viruses was detected (Figure 3C). Notably, GLuc activity was higher in d7-GLuc-infected cells compared to the cells infected with d8-GLuc (Figure 3C), probably due to the different expression levels of ORF7 and ORF8.

To examine whether the replacement of viral ORFs 6, 7a, and 8 by fluorescence reporters or the introduction of an additional EYFP gene between ORF8 and N impedes viral replication, we analyzed the growth kinetics in CaCo-2 cells by RT-qPCR (Figure 3D). We obtained similar replication curves for all the fluorescence reporter viruses compared to the WT. Only the full-length reporter virus after8-YFP, which expresses EYFP as an additional gene between ORF8 and N, showed a slightly decreased fitness at 24–48 hpi (Figure 3D). However, the peak viral titers were approximately the same for all the viruses tested, demonstrating that the replacement of viral ORFs or the introduction of an additional ORF does not critically influence viral replication. To evaluate differences in the reporter expression kinetics and expression levels of the distinct fluorescence viruses, we assessed their expression in infected CaCo-2 cells over a period of 72 h by fluorescence microscopy (Figure 3E, green signal). As a control, we stained the viral spike protein to detect infected cells (Figure 3E, red signal) and Hoechst 33342 (blue signal) to visualize the nuclei. Although all the reporter viruses tested within this study showed reporter expressions, we observed differences between the distinct variants. For d6-YFP (Figure S2) and d7-GFP (Figure S3), only a few reporter-expressing cells were detected compared to the cells that were stained positive for the spike protein at 12 hpi.

An increase in spike and reporter expression for both d6-YFP and d7-GFP over time was observed, which peaked at 48 hpi (Figures S2 and S3). At 72 hpi, we noticed a decline of positive cells and the total cell numbers, most likely due to detachment of the cells upon syncytia formation. In contrast, the GFP and spike signals in the cells infected with d8-GFP were already prominent at 12 hpi (Figure S4), increased over time, but also declined after 48 hpi. Notably, not only the GFP but, also, the spike and Hoechst signals were lost, indicating that cells infected with d8-GFP detach faster compared to those infected with d6-YFP or d7-GFP. Interestingly, we observed fewer YFP-positive cells after the infection of CaCo-2 cells with the full-length reporter virus after8-YFP (Figure S5), although the spike counterstaining indicates an expression pattern comparable to the d8-GFP reporter virus.

To further evaluate the stability of the different reporters, we investigated the presence of subgenomic (sg) RNAs of the respective reporters upon serial passaging on CaCo-2 cells using a RT-qPCR approach with probes specific for YFP/GFP or GLuc (Figure S6). Interestingly, we observed a decrease of sgRNAs transcribed from d8 viruses with an increasing cell culture passage, whereas the d6- and d7-reporter viruses remained stable over time. Similarly, the stability of the full-length reporter virus after8-YFP also diminished over time. Thus, ORF6 and ORF7 represent appropriate genomic positions for the insertion of reporters with constant expression levels, making them suitable for the following molecular applications.

Finally, we generated a recombinant SARS-CoV-2 marker virus that expresses 3xFLAG-tagged nucleoprotein (N-3xFLAG; Figure 3A). To analyze whether the insertion of the 3xFLAG tag impedes viral replication, we again infected CaCo-2 cells and determined the replication kinetics in comparison to the WT by RT-qPCR (Figure 3F). Although the tag may minimally reduce viral fitness, the marker virus nevertheless replicates in CaCo-2 cells with a growth curve comparable to the WT. To test if the 3xFLAG-tag is detectable upon infection, we performed a Western blot analysis of Vero E6 cells infected with the N-3xFLAG virus (Figure 3G). As a control, we used HEK293T cells that were transfected with an N-3xFLAG expression vector. As expected, we detected N-3xFLAG in the infected cells, demonstrating that this marker virus is a suitable tool for distinct biochemical assays like interaction studies or the microscopic detection of viral nucleocapsids.

### 2.4. Reporter-Based Confirmation of Antiviral Activity of Drugs

**Figure 4:**
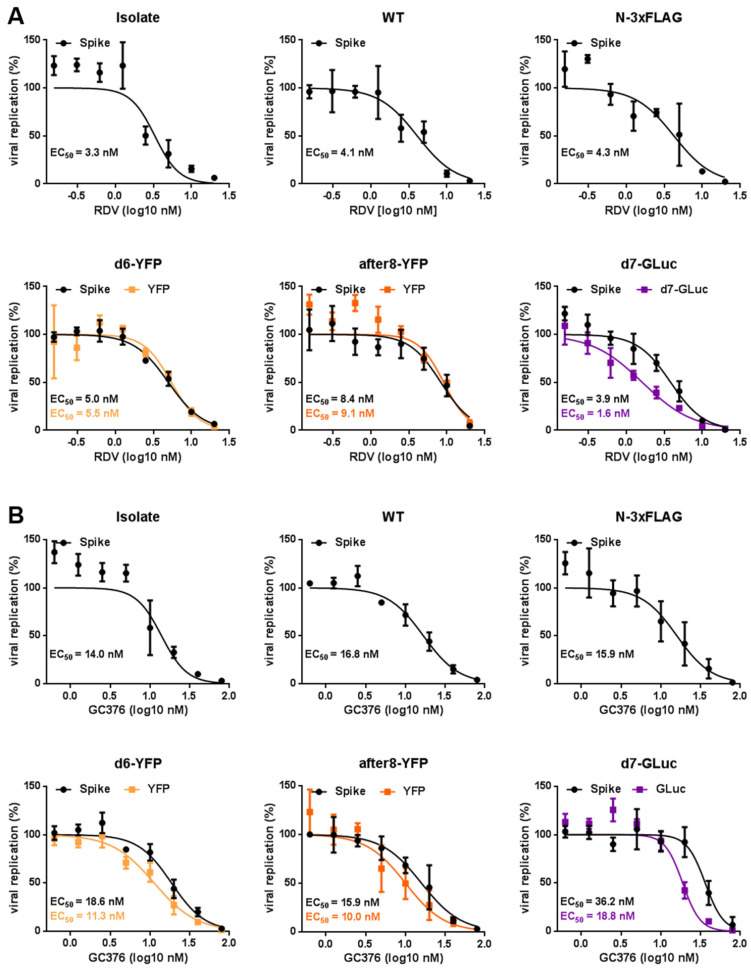
Dose response of the recSARS-CoV-2 reporter viruses to RDV and GC376. CaCo-2 cells were infected with the clinical isolate, the recSARS-CoV-2 WT, the N-3xFLAG marker virus, fluorescence reporter viruses expressing YFP instead of ORF6 (d6-YFP) or between ORF8 and N (after8-YFP), and the luciferase reporter virus d7-GLuc, which expresses gaussia luciferase instead of ORF7a (MOI = 0.005). At the same time, the cells were treated with 2-fold serial dilutions of remdesivir (RDV) (**A**) or GC376 (**B**). The cells were harvested at 30 hpi, and viral replication was determined by the quantification of spike expression (immunostaining with mAb against spikes), fluorescence reporter, or luciferase expression, respectively. The percentage of viral replication was determined relative to the solvent-treated cells. The 50% effective concentration (EC_50_) was assessed by nonlinear four-parameter curve fitting using GraphPad Prism and are shown within the respective graphs. Mean values of quadruplicates relative to the solvent-treated cells ± SD are depicted.

To assess the antiviral activity of the two reference drugs remdesivir (RDV) and GC376 against our recombinant (reporter) viruses, we conducted infection assays in CaCo-2 cells (Figure 4). The nucleoside analog RDV is a direct-acting, broad-spectrum antiviral that blocks the RNA-dependent RNA polymerase [12]. It was the first FDA-approved drug for emergency use to treat COVID-19. GC376, an inhibitor of the viral protease 3CL^pro^, also shows broad-spectrum antiviral activity against coronaviruses and potently inhibits SARS-CoV-2, at least in vitro [13,14]. Here, we compared the antiviral activity of RDV (Figure 4A) and GC376 (Figure 4B) against the clinical isolate, recSARS-CoV-2 WT, as well as the reporter viruses d6-YFP, after8-YFP, and d7-GLuc and the marker virus N-3xFLAG (Figure 4). As readouts, we either used the respective reporter (YFP for the fluorescence reporter viruses and gaussia luciferase activity for the GLuc reporter virus, respectively) or an in-cell fluorescence assay with which we stained the viral spike protein. To dissect antiviral activity from cytotoxicity, a Neutral Red Assay was performed (CC_50_ RDV = 72.7 µM, CC_50_ GC376 > 100 µM; 7). Importantly, the calculated EC_50_ values confirmed the inhibitory potential of both antivirals against SARS-CoV-2. Their antiviral activity was within a consistent range of EC_50_ values in the lower nanomolar range. Notably, the reporter readouts are also consistent and comparable to the signals obtained by spike antigen staining, even though we noticed minor biological variabilities, depending on the respective virus and particular assay used. Moreover, neither fusion of the 3xFLAG-tag to the nucleoprotein nor the introduction of an additional artificial ORF, as was the case in the after8-YFP full-length reporter virus, altered the antiviral activity of RDV and GC376. Taken together, the reporter viruses generated herein represent suitable tools for the time- and cost-efficient screening of antiviral drug candidates with eased handling.

### 2.5. Visualization of Incoming recSARS-CoV-2 Particles and Newly Synthesized Viral Genomes

**Figure 5:**
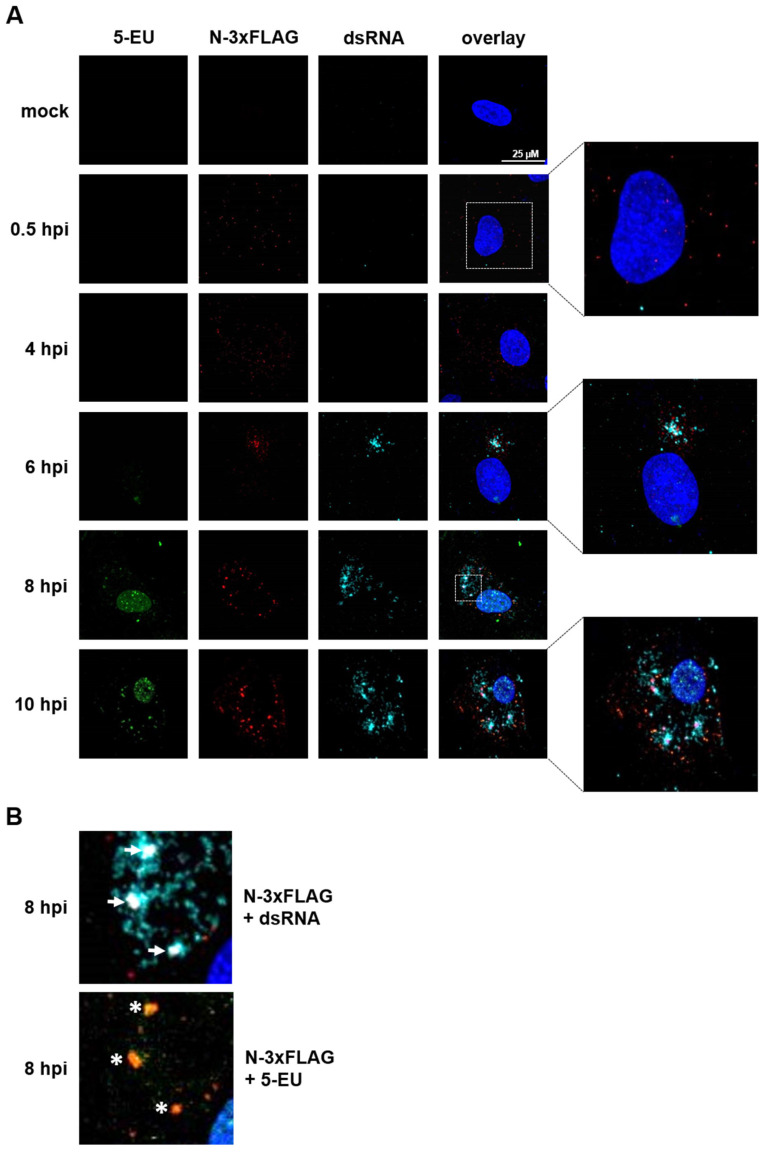
Visualization of the incoming SARS-CoV-2 nucleocapsids and newly synthesized viral RNA. (**A**,**B**) Vero E6 cells were infected with a recSARS-CoV-2 marker virus expressing a 3x-FLAG-tagged nucleoprotein (N-3xFLAG) under high MOI conditions (MOI~5). After one hour, the virus inoculum was replaced by a cell culture medium containing 20-µg/mL actinomycin D to inhibit cellular transcription. One hour later, 5-ethynyl uridine (5-EU) at a final concentration of 1 mM was added to the cells. The cells were fixed at the indicated time points post-infection, blocked, and permeabilized. A copper(I)-catalyzed alkyne-azide cycloaddition (CuAAC) reaction was performed with Alexa 488-conjugated picolyl azide to label a newly synthesized viral RNA (green). Afterwards, the cells were stained with antibodies against FLAG-tag to detect N-3xFLAG (red) and dsRNA to visualize the viral replication intermediates (cyan). Hoechst 33342 was used for nuclear staining (blue). The cells were analyzed by confocal laser scanning microscopy. Representative Z stacks and overlays are shown. (**B**) Enlargement of the overlapping signals (white dotted square in (**A**), 8 hpi). White arrows indicate overlapping signals for N-3xFLAG and dsRNA (visible as a white signal), white asterisks (*) imply merging signals of N-3xFLAG and dsRNA (visible as a yellow signal).

Finally, we used our newly generated recSARS-CoV-2 N-3xFLAG marker virus, which expresses 3xFLAG-tagged nucleoprotein (N), to visualize the incoming viral particles upon infection. Moreover, copper(I)-catalyzed alkyne-azide cycloaddition (CuAAC), also known as click chemistry, was applied to detect newly synthesized SARS-CoV-2 RNA. Vero E6 cells were infected with the N-3xFLAG marker virus at high MOI conditions (MOI ~5) and treated with actinomycin D to inhibit cellular transcription, as well as the modified nucleoside analog 5-ethynyl-uridine (5-EU), which is incorporated into nascent RNA, and were followed over a period of 10 h post-infection (hpi). In addition to the 3xFLAG-tag, we detected double-stranded (ds) RNA, an intermediate that naturally occurs during SARS-CoV-2 replication and, thus, indicates viral replication centers, by immunostaining. As early as 30 min post-infection, we sensitively detected viral particles as small, localized dots (Figure 5A, 0.5 hpi, red dots). Within the next few hours, the staining signal got more condensed (Figure 5A, 4 hpi and data not shown) and started to accumulate at the sites of viral replication (Figure 5A, 6 hpi). At this time point, viral replication was also clearly detectable by the staining of dsRNA (Figure 5A, 6 hpi, cyan), whereas newly synthesized RNA was not yet detectable by click chemistry. This might be due to weak signals as a result of insufficient 5-EU incorporation at 6 hpi. Notably, most of the signals for N-3xFLAG and dsRNA overlapped, demonstrating that our recombinant marker virus is suitable to probe viral replication centers. At 8 hpi, the cells start to massively produce new viral particles, as shown by the increase in N-3xFLAG signal throughout the entire cell, and viral transcripts, indicated by the abundant dsRNA signal (Figure 5A, 8 hpi and Figure 5B, white arrows). Remarkably, at this time point, the signals for newly synthesized RNA could also be visualized by 5-EU detection (Figure 5A, 8 hpi, green signal). Despite the actinomycin D treatment, background 5-EU signals for cellular transcription were also observed in the cell nucleus. However, signals specifically representing SARS-CoV-2 RNA can be recognized by merging the signals with N-3xFLAG staining (Figure 5B, white asterisks). These signals increase in intensity as the replication proceeds (Figure 5A, 10 hpi). Taken together, we successfully used our Lambda Red recombination-based mutagenesis approach to generate a SARS-CoV-2 marker virus that allows the tracking of viral particles and the detection of viral replication centers on the single-cell level. Last, but not least, we utilized dsRNA staining and click chemistry to visualize the newly synthesized viral RNA.

## 3. Discussion

Reverse genetics approaches to generate recombinant viruses are a powerful tool to characterize newly emerged viruses. Although several reverse genetics systems for CoVs, and especially SARS-CoV-2, have been described, those existing systems have several labor- and cost-intensive, error-prone, and time-consuming drawbacks, including the chemical synthesis of large DNA fragments [9], in vitro ligation [3], and in vitro transcription [3,4]. Here, we established a BAC-based reverse genetics system by applying Red recombination commonly used in the context of large DNA viruses, particularly herpesviruses. Of note, the technique can avoid the necessity of a previous viral isolate or allow to obtain clones from diagnostic samples in virus-inactivating chaotropic buffers. The advantage of this approach is the fast and easy manipulation of viral sequences by homologous recombination. By using this method, we successfully generated SARS-CoV-2-encoding BACs and reconstituted an infectious virus upon the transfection of HEK293T cells. Those cells allow the application of conventional, cost-efficient, well-established standard transfection methods with high transfection rates, an approach that has also been used by others [6]. After passaging the supernatant onto susceptible cells, we obtained a recombinant virus with comparable replication characteristics and similar peak virus titers compared to a clinical isolate. Moreover, we successfully applied our recombination technique to generate a set of different reporter viruses. The replacement of viral ORF7a or ORF8 with either EGFP or gaussia luciferase (GLuc) and ORF6 with EYFP led to reporter viruses that display WT-like replication kinetics and peak viral titers. This demonstrates that the deletion of either ORF by GLuc or EGFP/EYFP does not affect the viral fitness and that those ORFs are dispensable for SARS-CoV-2 replication in cell cultures. In contrast to ORF7a-deficient mutants reported by other groups, which showed impaired replication fitness compared to the WT strain [3,4,6], our newly generated d7-GFP and d7-GLuc reporter viruses displayed WT-like growth kinetics in Caco-2 cells, indicating that ORF7a is dispensable in vitro.

When we visually compared cells infected with the distinct reporter viruses, we observed differences in the fluorescence reporter expression profiles and kinetics, depending on the subgenomic localization of the reporter gene. This discrepancy might be explained by variations in the expression kinetics of individual viral proteins. Thus, it might be necessary to choose different time points for fluorescent readouts, depending on the viral strain that is used. Moreover, our results point towards a decreased genomic stability of viruses that express reporter genes instead of ORF8 compared to ORF6 or ORF7 over longer passaging in cell cultures. The reduced stability of d8 constructs indicating a lower genomic stability might be due to the replacement of a fast-evolving area of the SARS-CoV-2 genome [15]. Deletions or frameshift mutations in SARS-CoV-2 ORF8 have been observed at the beginning of the pandemic [16], in the recent B.1.1.7 (Alpha) variant, and, correspondingly, in the SARS-CoV ORF8 in the late SARS-CoV epidemic [17,18,19]. Furthermore, it might be possible that the introduction of an additional artificial ORF, like is the case in our full-length after8-YFP reporter virus, is also not stable over time. If this observation is also related to the apparent instability of the ORF8 region requires further investigation. However, since all reporter viruses are stable over time periods that are usually used for standard molecular approaches, all reporter viruses generated within this study are well-suited for the assays applied herein.

Virus-encoded fluorescent reporters allow the direct and highly quantitative detection of viral replication, as well as imaging approaches, which ease the testing of antiviral drug candidates. Here, we showed that our reporter readouts yielded similar EC_50_ values for the reference compounds RDV and GC376 compared to the immunostaining of the spike protein. Thus, without the requirement of additional detection reagents like antibodies or nucleic acid extraction followed by RT-qPCR, they provide a time-saving and cost-efficient means of analyses. Based on the available instrumentation present in the individual BSL-3 facilities, different types of reporters like the GLuc-expressing variants described herein can be designed to meet those requirements, thus further accelerating antiviral screenings. In particular, the use of secreted GLuc enables the analysis of different time points from the same sample without the need for cell lysis, thus leaving infected cells intact for additional confirmation or assays.

Moreover, we generated the full-length reporter virus after8-YFP with an additionally introduced artificial ORF encoding EYFP between ORF8 and N. Since this virus shows WT-like replication, this indicates that the plasticity of the SARS-CoV-2 genome tolerates the insertion of an additional ORF. The reduced level of reporter expression compared to the reporter introduced by gene replacement may reflect the TRS usage and may, in future experiments, be optimized by altering this element to increase the generation of reporter-specific sgRNAs. Possibly, the insertion of a second artificial ORF might be feasible, opening the potential of generating dual-reporter viruses encoding both fluorescence and luminescence reporters without the need to replace any viral protein. Thus, such recombinant viruses are prerequisite tools to study SARS-CoV-2 biology in vivo.

Furthermore, this methodological approach is not restricted to the examples presented herein but provides countless possibilities for generating functional knockouts of the versatile SARS-CoV-2 enzymes, the analysis of different spike alleles from circulating variants of concern (VoC), their contribution to immune and vaccine escape [20,21], and their relevance for virus transmission and pathogenicity, as well as the characterization of potential drug-resistance mutations [22,23] in an otherwise isogenic background. Moreover, our approach enables the rapid deletion of viral proteins to gain more insights into their contributions to replication, pathogenesis, transmission, and disease outcome in an infectious background. Moreover, the introduction of single restriction sites by Red recombination allows the directed integration and replacement of any desired region within the viral genome by techniques such as Gibson assembly [24].

Last, but not least, we cloned a full-length marker virus that expresses 3xFLAG-tagged nucleocapsid, which allows the detection of viral nucleocapsids on the single-cell level. Thus, to our knowledge, we were the first that applied the tagged SARS-CoV-2 N protein for visualization by immunofluorescence approaches, which provides the advantage of specificity and background minimization. In combination with biorthogonal click chemistry to label viral RNAs and/or the detection of dsRNA, these methods facilitate a spatiotemporal analysis of the viral replication processes, as well as the influence of host and/or viral immune regulators.

In summary, by introducing BAC-based Red recombination into SARS-CoV-2 research, we developed a powerful, reliable, and convenient platform to facilitate studies answering numerous questions concerning the biology of SARS-CoV-2. Since CoVs are prone to transmission to new host species, gaining more insights into SARS-CoV-2 replication, pathogenicity, transmission, and interaction with host cell factors leads to a better preparedness for future pandemic CoVs.

## 4. Materials and Methods

### 4.1. Biosafety

All infection experiments were performed under biosafety level (BSL)-3 conditions. Modifications and preparations of pBSCoV2 BACs containing the full-length SARS-CoV-2 genome were conducted under BSL-2 conditions.

### 4.2. Cell Culture

In general, all the cell lines were cultivated at 37 °C, 5% CO_2_, and 80% humidity. All the cell lines were tested frequently for mycoplasma contaminations using the MycoAlert™ Mycoplasma Detection Kit (LT07-703, Lonza, Basel, Switzerland) according to the supplier’s manual. Human embryonic kidney (HEK) 293T (ATCC^®^ CRL-3216™, ATCC, Manassas, VA, USA) and Vero E6 cells (85020206, Sigma-Aldrich, St. Louis, MO, USA) were maintained in Dulbecco’s Modified Eagle’s Medium (DMEM; 11500516, Thermo Fisher Scientific, Waltham, MA, USA) supplemented with 10% heat-inactivated fetal calf serum (FBS-12A; Capricorn Scientific, Ebsdorfergrund, Germany), 2-mM GlutaMAX™ (35050061, Thermo Fisher Scientific), 25-mM HEPES (15630080, Thermo Fisher Scientific), and 50-µg/mL gentamycin (1405-41-0, Serva Electrophoresis, Heidelberg, Germany). The CaCo-2 cells (kindly provided by Konstantin Sparrer, University Hospital Ulm, Germany) were cultivated in DMEM additionally containing 1x MEM Non-Essential Amino Acids Solution (11140050, Thermo Fisher Scientific). The HEK293T-ACE2 and HEK293T-T7RNAP/N cells were maintained in DMEM additionally supplemented with 5-µg/mL blasticidin (asnt-bl-1, InvivoGen, San Diego, CA, USA) or 5-µg/mL blasticidin and 2-µg/mL puromycin (SC-1080713, Santa Cruz Biotechnology, Dallas, TX, USA), respectively. Stable HEK293T-ACE2 cells were generated by lentiviral transduction with pLV-EF1a-IRES-Blast (#85133, Addgene, Watertown, MA, USA) encoding the human ACE2 gene. HEK293T-T7RNAP/N cells were established using the lentiviral vectors pLV-EF1a-IRES-Blast and pLV-EF1a-IRES-Puro (#85132, Addgene, Watertown, MA, USA) encoding the T7 RNA polymerase or the SARS-CoV-2 nucleoprotein (N), respectively.

### 4.3. Plasmids

For the cloning of pLV-EIB-N and pLV-EIB-ACE2, the pLV-EF1a-IRES-Blast vector (#85133, Addgene, Watertown, MA, USA) was used. For the cloning of pLV-EIP-T7RNAP, we used the pLV-EF1a-IRES-Puro vector (#85132, Addgene). The viral nucleoprotein (N) was amplified from the patient material, T7 RNA polymerase (T7RNAP) was amplified from pCAGT7 (kindly provided by Marco Thomas, Institute for Clinical and Molecular Virology, Erlangen, Germany), and pCG1-hACE2 [25] served as the template for the amplification of hACE2. All the vectors were generated by Gibson assembly [24] and confirmed by Sanger sequencing and a Western blot analysis. For cloning pLV-EF1a-IRES-Blast-N-3xFLAG, the pBSCoV2-N-3xFLAG bacmid generated within this study was used as the template. All the primers used for plasmid generation are listed in Table S1 and were purchased from Integrated DNA Technologies (IDT, Coralville, IA, USA).

### 4.4. Western Blot Analysis

For the Western blot analysis, pelleted cells were lysed in RIPA buffer (1% Triton X-100, 0.5% sodium deoxycholate, 0.1% SDS, 150-mM NaCl, 1-mM EDTA, and 50-mM Tris (pH 8.0)) with freshly added aprotinin (A2132, AppliChem, Darmstadt, Germany) and leupeptin (A2183, AppliChem). The protein content of the soluble fraction was assayed with a Pierce™ BCA Protein Assay Kit (23225, Thermo Fisher Scientific). The samples were diluted in a Laemmli SDS sample buffer, separated by SDS-PAGE, and the proteins were transferred on PVDF membranes (Immobilon^®^-P (IPVH00010) for the HRP-labeled secondary antibodies and Immobilon^®^-FL (IPFL00010) for the fluorescence-labeled secondary antibodies, both from Merck Millipore, Burlington, MA, USA). The membranes were blocked with 5% dry milk powder prior to antibody incubation. For the detection of T7-RNA polymerase, a mouse monoclonal antibody (70566, Novagen^®^/Sigma-Aldrich, St. Louis, MO, USA) at a dilution of 1:5000 was used with the HRP-coupled anti-mouse secondary antibody. The viral nucleoprotein was stained with a rabbit polyclonal nucleocapsid (N) antibody (ARC2372, Thermo Fisher Scientific) at a dilution of 1:2000 and an anti-rabbit-Alexa555 secondary antibody. Human ACE2 was probed with a mouse monoclonal antibody (ACE2 E-11, Santa Cruz Biotechnology) at 1:500 and an Alexa647-labled anti-mouse secondary antibody. The 3x-FLAG-tagged nucleoprotein was detected with a rabbit polyclonal anti-DYKDDDDK tag antibody (PA1-984B, 1:1000; Thermo Fisher Scientific).

### 4.5. Cloning of Full-Length SARS-CoV-2 Genome

For the cloning of a passage-free SARS-CoV-2 genome, an anonymized residual sample from a patient with SARS-CoV-2 infection was used as a template. The total nucleic acids of a respiratory swab sample were extracted on an automated Qiagen EZ1 analyzer (Qiagen, Hilden, Germany) using the Qiagen EZ1 virus mini kit v2.0 (Qiagen, 955134) according to the manufacturer’s instructions. The extracted nucleic acids were eluted in 90-µL ultrapure water. For cDNA synthesis, the extracted nucleic acids were reverse-transcribed with the LunaScript^®^ RT SuperMix Kit (E3010, New England Biolabs (NEB), Frankfurt am Main, Germany) according to the supplier’s manual. Afterwards, 5 U of RNaseH (M0297, NEB) were added to the mix, followed by incubation for 30 min at 37 °C and further dilution by the addition of 80 µL 0.1× TE buffer. The obtained cDNA was used for the amplification of four fragments covering the entire viral genome using Q5-DNA-Polymerase (NEB). The resulting fragments were assembled with the modified and *Pac*I-linearized pBeloBAC11 (Gene Bank Accession U51113, NEB) backbone pBeloCoV. For the construction of pBeloCoV, the CMV and T7 promoters, as well as the bGH polyA tail, were amplified from a pcDNA4 vector (V86320, Thermo Fisher Scientific). Gene blocks encoding for the hepatitis virus D ribozyme, the 5′ and the 3′ end of the SARS-CoV-2 genome with a flanking *Pac*I site, and a linker for the polyA tail were ordered from Integrated DNA Technologies (IDT, Coralville, IA, USA). The assembly of these modules using the NEBuilder HiFi DNA Assembly Cloning Kit (E5520, NEB) resulted in the modified pBeloCoV bacmid. The four fragments covering the SARS-CoV-2 genome were then assembled with the *Pac*I-digested pBeloCoV vector according to the NEBuilder HiFi DNA Assembly Cloning Kit protocol (E5520, NEB) to obtain pBelo-SARS-CoV-2 (pBSCoV2). After electroporation into *E. coli* NEB^®^ 10-beta (C3019, NEB), the clones were screened by restriction digestion and further analyzed by next-generation sequencing. To receive the clones with a full-length SARS-CoV-2 genome, one clone that was found to lack fragment 2 was transformed into the *E. coli* strain GS1783, and Red recombination was performed to insert the missing fragment, as previously described [10,11] (Figure S1). First, a kanamycin resistance cassette with short-sequence duplications to the SARS-CoV-2 genome at the site of recombination and flanking *Asc*I sites was PCR-amplified (7107-fwd-recAsc and 13997-rev-recAsc) using pEPkan-S as a template [11] and digested with *Dpn*I. The purified PCR product was electroporated into *E. coli* GS1783 containing the incomplete clone, and recombination was initiated. The positive clones that grew on kanamycin-containing plates were screened by colony PCR and restriction digestion. Purified DNA of two clones was digested with *Asc*I to remove the kanamycin resistance cassette, and afterwards, an assembly with the lacking fragment 2 was performed according to the NEBuilder HiFi DNA Assembly Cloning Kit protocols (E5520, NEB). The DNA was transformed into *E. coli* GS1783, and the resulting colonies were screened by restriction digestion. Clones with restriction patterns matching the pBSCoV2 bacmid with a full-length SARS-CoV-2 genome were confirmed by next-generation sequencing.

### 4.6. Generation of SARS-CoV-2 Reporter Viruses by Red-Mediated Recombination

To generate different SARS-CoV-2 marker and reporter viruses, the two-step markerless Red recombination system was used [10,11]. Within this study, pBSCoV2-N-3xFLAG (N-3xFLAG), deltaORF7a-GLuc (d7-GLuc), deltaORF7a-EGFP (d7-GFP), deltaORF8-GLuc (d8-GLuc), deltaORF8-EGFP (d8-GFP), deltaORF6-EYFP (d6-YFP), and after8-EYFP (after8-YFP) were cloned. All ultramer primers used for their assembly are listed in Table S1 and were purchased from IDT. As a starting point, the *E. coli* strain GS1783 containing the pBSCoV2 bacmid encoding the full-length SARS-CoV-2 genome was used. First, the kanamycin resistance cassettes for the first Red recombination were generated, with either one I-*Sce*I (for N-3xFLAG, d6-YFP, and after8-YFP) or two flanking *Sac*II restriction sites (for d7-Luc, d7-GFP, d8-Luc, and d8-GFP), as well as flanking sequences with homology to the corresponding site of insertion within the SARS-CoV-2 genome. For d6-YFP and after8-YFP, the pEP-EYFP-in vector [10] was used as a template, whereas pEPkan-S served as the template for N-3xFLAG and d7 or d8. Upon electroporation of the purified kanamycin cassettes into *E. coli* GS1783 containing pBSCoV2, kanamycin-resistant clones were screened by colony PCR and restriction digestion. In the case of N-3xFLAG, d6-YFP, and after8-YFP, the kanamycin resistance cassette was removed by in vivo cleavage upon the induction of I-*Sce*I with 1% arabinose, followed by a second Red recombination. For the d7 and d8 reporter viruses, the kanamycin resistance cassette was removed by digestion with *Sac*II. Assembly with the NEBuilder HiFi DNA Assembly Cloning Kit (E5520, NEB), according to the supplier’s protocols, inserted DNA fragments encoding for either gaussia luciferase (GLuc) or EGFP. Reporter cassettes were PCR-amplified with primers with homology to the site of insertion. Assembled bacmids were transformed again into *E. coli* GS1783. Colonies that lost the ability to grow on kanamycin-containing plates were analyzed by restriction digestion and Sanger sequencing of the recombination sites and inserts, as well as next-generation sequencing of the confirmed positive clones.

### 4.7. Next-Generation Sequencing and Computational Analysis

For library preparation, 50-ng purified bacmid DNA and the Nextera DNA Library Preparation Kit (FC-131.1024, Illumina, San Diego, CA, USA) were used. Tagmented DNA was amplified with specific dual index primers (Integrated DNA Technologies) using the NEBNext^®^ HiFi 2× PCR Master Mix (M0541, NEB) according to the manufacturer’s protocols. The libraries were cleaned up with AMPure XP beads (A63881, Beckman Coulter, Brea, CA, USA) and quantified using the Qubit dsDNA HS Assay Kit (Q32851, Thermo Fisher Scientific). Normalized, pooled libraries were analyzed by paired-end next-generation sequencing using a MiSeq reagent kit v2, with 300 or 500 cycles on a MiSeq™ Instrument (Illumina). The sequences were analyzed with CLC Genomics Workbench 21 (Qiagen Aarhus A/S, Aarhus, Denmark).

### 4.8. Virus Recovery

For virus reconstitution, a coculture of HEK293T cells stably expressing either ACE2 or the viral N protein and T7-RNA polymerase was transfected with 8 µg of purified pBelo-S-CoV-2 and 2 µg of pLV-EF1a-IRES-Blast-N using 20-µL GenJet™ Reagent (II) (SL100489, SignaGen^®^ Laboratories, Frederick, MD, USA) according to the manufacturer’s protocols in T25 tissue culture flasks. Three days post-transfection, the supernatant (P0) was transferred on CaCo-2 or Vero E6 cells for passage 1 (P1) virus stocks. Passage 2 (P2) virus stocks were obtained after the infection of CaCo-2 cells with 1:50 volume of the P1 virus. The viral titers were calculated by endpoint titration (TCID_50_). Positive wells were determined by in-cell immunostaining of the viral spike protein.

### 4.9. In-Cell Immunostaining

For the visualization of intracellular viral antigens, paraformaldehyde-fixed cells were permeabilized and blocked with 1% BSA and 0.2% Triton X-100 in PBS overnight at 4 °C or 1 h at RT. Afterwards, the cells were stained with a mouse monoclonal antibody against the viral spike protein (mAB-S TRES-VI6.18 [26], 1:2000 diluted in 1% BSA and 0.2% Triton X-100 in PBS) and Alexa488- or Alexa647-conjugated secondary antibodies (A11029/A31571, 1:2000 diluted in 1% BSA and 0.2% Triton X-100 in PBS, Thermo Fisher Scientific), respectively. In parallel, Hoechst 33342 (14533, Sigma-Aldrich) 1:5000 diluted in PBS served as the internal control for estimating the cell counts. To quantitatively evaluate the stained plates, a Victor X4 multilabel reader (Perkin Elmer, Waltham, MA, USA) was used. An ImmunoSpot^®^ S6 ULTIMATE UV Image Analyzer (Cellular Technology Limited/CTL, Shaker Heights, OH, USA) was used for visualization of the total cell numbers and infected cells.

### 4.10. RT-qPCR

CaCo-2 cells were seeded in 96-well plates. The next day, the cells were infected with the corresponding virus at an MOI of 0.005. The supernatants were harvested at 0, 12, 24, 36, 48, and 72 h post-infection. To quantify the viral genome copies, the supernatants were diluted 1:10 with H_2_O and heat-inactivated for 15 min at 95 °C. Prior to RT-qPCR, the viral supernatants were digested with proteinase K (final concentration of 0.136 mg/mL; PCR-grade, 3115828001, Sigma-Aldrich) for 1 h at 56 °C, followed by heat inactivation for 5 min at 95 °C. For the subsequent analysis, volumes of 5 µL of the digested supernatants were used, and the RT-qPCR was performed according to the Luna^®^ Universal Probe One-Step RT-qPCR Kit protocol (E3006, NEB) in an Applied Biosystems 7500 Real-Time PCR system (Applied Biosystems, Waltham, MA, USA). The primer and probe sequences targeting the viral polymerase (RdRp) were adapted and modified from Reference [27]. The probe was 5′-labeled with VIC (2′-chloro-7′phenyl-1,4-dichloro-6-carboxy-fluorescein) and 3′-labeled with BMN535 quencher modification (Biomers, Ulm, Germany).

### 4.11. Luciferase Assay

To measure the gaussia luciferase (GLuc) activity, the supernatants were heat-inactivated for 15 min at 95 °C and cooled down at RT prior to the luciferase assay. Subsequently, 50 µL of a 1:40 dilution were transferred into a white 96-well plate. Per well, 100-µL Renilla assay buffer (1.1-M NaCl, 2.2-mM Na_2_EDTA, and 0.22-M KPO_4_) and 0.2 µL of 0.7-mM Coelenterazine (102171, PJK Biotech, Kleinblittersdorf, Germany) were used. The luciferase activity was determined in an Orion II Microplate Luminometer (Berthold Detection Systems, Bad Wildbad, Germany) with a 4 s delay time and 5 s measurement time.

### 4.12. Antiviral Compounds and EC_50_ Calculation

RDV (Gilead, Foster City, CA, USA) and GC376 (T5188, TargetMol, Boston, MA, USA) were used as the reference compounds to determine the anti-SARS-CoV-2 activity for recSARS-CoV-2. The CaCo-2 cells were treated with 2-fold serial dilutions of either RDV (20 nM–0.16 nM) or GC376 (80 nM–0.63 nM) and infected with different SARS-CoV-2 strains at an MOI of 0.005. At 30 hpi, the cells were fixed and analyzed. The infection rates were assessed by immunostaining of the viral spike and reporter expression, respectively. The percentage of viral replication was determined relative to the solvent-treated cells. The 50% effective concentration (EC_50_) was assessed by nonlinear four-parameter curve fitting using GraphPad Prism 6 (GraphPad Software, San Diego, CA, USA).

### 4.13. Neutral Red Assay

The cytotoxicity of RDV and GC376 was determined by the neutral red uptake assay (N-2889, Sigma-Aldrich). The CaCo-2 cells were seeded in 96-well plates and incubated with the antiviral compounds for 3 days. Staurosporine (AG-CN2-0022-M005, Biomol, Hamburg, Germany) at a concentration of 10 µM was used as the toxicity control. The assay was performed as previously described [28] using a final concentration of 40-µg/mL neutral red. The amount of incorporated neutral red was quantitated in a Victor X4 multilabel reader (Perkin Elmer LAS GmbH, Rodgau, Germany) by fluorescence measurements at 560/630 nm.

### 4.14. Click Chemistry and Immunofluorescence Staining

Vero E6 cells were seeded in 24-well plates on round coverslips. The day after, the cells were infected with pBSCoV2-N-3xFLAG. One hour post-infection (hpi), the virus inoculum was removed, and the cells were incubated in a medium containing 20-µg/mL actinomycin D (CAS 50-76-0, Santa Cruz Biotechnology). After one additional hour, a 100-µL medium containing 20-µg/mL actinomycin D and a final concentration of 1-mM 5-ethynyl uridine (5-EU) (CLK-N002-10, Jena Bioscience, Jena, Germany) were added to the 2, 4, 6, 8, and 10 hpi time points. The cells were harvested at 0.5, 1, 2, 4, 6, 8, and 10 hpi, respectively; washed with PBS; and fixed with 4% paraformaldehyde in PBS for 20 min at RT. After washing with PBS, the cells were blocked and permeabilized with 1% BSA and 0.2% Triton X-100 in PBS overnight at 4 °C. To detect the incorporated 5-EU, a copper(I)-catalyzed alkyne-azide cycloaddition (CuAAC) reaction (also known as click chemistry) was performed. Therefore, the cells were incubated for 30 min with 1-mM CuSO_4_, 1-mM THPTA (CLK-1010, Jena Bioscience), 1-µM AF488-Picolyl-Azide (CLK-1276, Jena Bioscience), 10-mM sodium ascorbate, and 10% DMSO at RT in PBS in the dark. The cells were incubated with primary and secondary antibodies diluted in 1% BSA and 0.2% Triton X-100, with three washing steps with 0.1% Tween-20 in PBS after each antibody incubation. The viral nucleoprotein (N-3xFLAG) was stained with a rabbit polyclonal anti-DYKDDDDK tag antibody (PA1-984B, 1:1000; Thermo Fisher Scientific) and an anti-rabbit-Alexa555 antibody (A31572, 1:1000, Thermo Fisher Scientific). Double-stranded viral RNA (dsRNA) replication intermediates were stained with a monoclonal anti-dsRNA J2 antibody (IgG2a, RNT-SCI-10010200, 1:000; Jena Bioscience) and an anti-mouse IgG2a-Alexa647 antibody (A21242, 1:1000, Thermo Fisher Scientific). Hoechst 33342 (14533, 1:1000, Sigma-Aldrich) was used for the detection of cellular DNA. The coverslips were mounted on microscope slides in ProLong™ Glass Antifade Mountant (P36982, Thermo Fisher Scientific). The cells were visualized by confocal laser scanning microscopy (Leica TCS SP5 equipped with a 63x1.4 HCX PL APO CS oil immersion objective; Leica Microsystems, Wetzlar, Germany) and Leica Application Suite Advanced Fluorescence (Leica Microsystems); the obtained pictures were minimally processed with Adobe^®^ Photoshop Elements 15 (Adobe, San Jose, CA, USA).

### 4.15. Reporter Stability Assay

CaCo-2 cells were seeded in a 12-well format and infected with P2 SARS-CoV-2 variants d6-YFP, d7-GFP, d7-Luc, d8-GFP, or after8-YFP at 1:2000 dilution. The viruses were passaged every two days on freshly seeded CaCo-2 cells, and the subgenomic RNA was isolated using TRI Reagent (R2050-1-50, Zymo Research, Freiburg, Germany) according to the manufacturer’s protocols. Five microliters of isolated RNA were utilized for the RT-qPCR. Therefore, the Universal Probe One-Step RT-qPCR kit (NEB) was used according to manufacturer’s instructions. For detection, RdRp-VIC, Luc-FAM, and YFP/GFP-FAM probes were used (Table S1).

## Supplementary Materials

The following are available online at www.mdpi.com/article/10.3390/ijms221910188/s1.
