# Supplementary material for: Cloning of a Passage-Free SARS-CoV-2 Genome and Mutagenesis Using Red Recombination
Source: Int J Mol Sci. 2021 Sep 22;22(19):10188. doi: 10.3390/ijms221910188 (PMC8507965; doi:10.3390/ijms221910188)
Supplement: Supplementary file 1 [file ijms-22-10188-s001.zip › ijms-1364512-supplementary.pdf]

International Journal of *Molecular Sciences*

MDPI

Supplementary Materials

Cloning of a passage-free SARS-CoV-2 genome and mutagenesis using Red recombination

Alexandra Herrmann [1], Doris Jungnickl [1], Arne Cordsmeier[1], Antonia Sophia Peter[1], Klaus Überla[1], and Armin Ensser [1,*]

Table S1. Oligonucleotides used within this study.

Application	Name	Sequence (5' → 3')
pLV-EF1a-IRES-Blast-N	LVfwdN	gtcgtgaggatccgcacagtggcggccgccgccaccatgtctgataatggaccccaaaat
	LVrevN	tgctagaggctgatcagcgggtttaaacttaggcctgagttgagtcagcactg
pLV-EF1a-IRES-vector	LV-IRES-Not-Kozak-Atg	catggtggcggcggccgccactgtgcggatcctcacgacacctgaaatg
	FLAG-Stop-Pme	gattacaaggatgacgacgataagtgagtttaaacccgctgatcagcctc
pLV-EF1a-IRES-Blast-ACE2	LVfwdACE2	gtcgtgaggatccgcacagtggcggccgccgccaccatgtcaagctcttcctggctcctt
	LVrevACE2	tgctagaggctgatcagcgggtttaaacctaaaaggaggtctgaacatcatca
pLV-EF1a-IRES-Puro-T7RNAP	T7-LVfwd	gtcgtgaggatccgcacagtggcggccgccgccaccatgaacacgattaacatcgctaag
	T7-LVrev	tgctagaggctgatcagcgggtttaaactcacgcgaacgcgaagtccgactctaag
pLV-EF1a-IRES-Blast-N-3xFLAG	LVfwdN	gtcgtgaggatccgcacagtggcggccgccgccaccatgtctgataatggaccccaaaat
	asFLAGtag	cttatcgtcgtcatccttgtaatc
Gene blocks (IDT)	Gaussia Luc	atgggagtcaaagttctgtttgccctgatctgcatcgctgtggccgaggccaagcccaccgagaacaacgaagacttcaacatcgt ggccgtggccagcaacttcgcgaccacggatctcgatgctgaccgcgggaagttgcccggcaagaagctgccgctggaggtgct caaagagatggaagccaatgcccggaaagctggctgcaccaggggctgtctgatctgcctgtcccacatcaagtgcacgcccaa gatgaagaagttcatcccaggacgctgccacacctacgaaggcgacaaagagtccgcacagggcggcataggcgaggcgatc gtcgacattcctgagattcctgggttcaaggacttggagcccatggagcagttcatcgcacaggtcgatctgtgtgtggactgcaca actggctgcctcaaagggcttgccaacgtgcagtgttctgacctgctcaagaagtggctgccgcaacgctgtgcgacctttgccag caagatccagggccaggtggacaagatcaagggggccggtggtgactaa
	CoVends-PacI	ttaaaggtttataccttcccaggtaacaaaccaaccaactttcgatctcttgtagatctgttctctaaacgaactttaaaatctgtgtggc tgtcactcggctgcatgcttagtgcactcacgcagtataattaataactaattactgtcgttgacaggacacgagtaactcgtctatctt ctgcaggctgcttacggtttcgtccgtgttgcagccgatcatcagcacatctaggtttcgtccgggtgtgaccgaaaggtaagatgg agagccttgtccctggtttcaacgagaaaacacacgtccaactcagtttgcctgttttacaggttcgcgacgtgctcgtacgttaatta aatgggctatataaacgttttcgcttttccgtttacgatatatagtctactcttgtgcagaatgaattctcgtaactacatagcacaagta gatgtagttaactttaatctcacatagcaatctttaatcagtgtgtaacattagggaggacttgaaagagccaccacattttcaccgag gccacgcggagtacgatcgagtgtacagtgaacaatgctagggagagctgcctatatggaagagccctaatgtgtaaaattaatt ttagtagtgctatccccatgtgattttaatagcttcttaggagaatgacaaaaaaaaaa
Cloning pBeloCoV	BeloCMV-F	cattatacgaagttatattcgatgcggccgctagtaatcaattacggggtcattag
	CMV-T7-CoV-R	cctgggaaggtataaacctttaatctccctatagtgagtcgtattaat

	bGHpA-Belo-F	tctagttgccagccatctgttgtt
	bGHpA-Belo-R	atgagaattggtcgacggcccgggcggccgcccccctagagccccagctggttctttc
	HDV Ribozyme	ggccggcatggtcccagcctcctcgctggcgccggctgggcaacattccgaggggaccgtcccctcggtaatggcgaatgggac
	Linking-pA-F	cttaggagaatgacaaaaaaaaaaaaaaaaaaaaaaaaaaaaaaggccggcatggtcc
	Linking-pA-R	ggaccatgccggccttttttttttttttttttttttttttttttgtcattctcctaag
Fragment 1	326-Fwd	ttacaggttcgcgacgtgctcgtacgtggctttggagactccgtggag
	7150-Rev	taaaccactaagacaaacactacaagg
Fragment 2	7127-Fwd	tgtagtgtttgtcttagtggtttag
	13975-Rev	ctaagttggcgtatacgcgtaatatatc
Fragment 3	13959fwd	cgtatacgccaacttaggtgaac
	21192-Rev	agcattccaagaatgttctgttat
Fragment 4	21169-Fwd	ataacagaacattcttggaatgct
	29581-ORF10-Rev	agcgaaaacgtttatatagcccatctg
Insertion fragment 2	7107fwd-recAsc	actgtactggttctataccttgtagtgtttgtcttagtggtttaggcgcgccaaccaattaaccaattctgattag
	13934fwd-recAsc	ttgtagaaaacccagatatattacgcgtatacgccaacttaggtggcgcgccaaccaattaaccaattctgattag
N-3xFLAG	N3F-in-Fwd	tccaaacaattgcaacaatccatgagcagtgctgactcaactcaggccgactacaaggaccacgatggtg
	N3F-in-Rev	aacgtttatatagcccatctgccttgtgtggtctgcatgagtttacttatcgtcgtcatccttgtaatcgatgtcatgatctttataacaac caattaaccaattctgattag
d7-GLuc	GLuc-dORF7a-Fwd	aatggagattgattaaacgaacatgggagtcaaagttctgtttgcc
	GLuc-dORF7a-Rev	gaagtcaattaatgaaagttcaatcagtcaccaccggccccccttgatc
	27351fwd-recSac-dORF7a	tcaattagatgaagagcaaccaatggagattgattaaacgaacatgccgcggaaccaattaaccaattctgattag
	27801rev-recSac-dORF7a	aggctaaaaagcacaaatagaagtcaattaatgaaagttcaatcaccgcggaggatgacgacgataagtaggg
d7-GFP	EGFP-dORF7a-Fwd	atggagattgattaaacgaacatggtgagcaagggcgaggag
	EGFP-dORF7a-Rev	agtcaattaatgaaagttcaatcacttgtacagctcgtccatgccg
	27351fwd-recSac-dORF7a	tcaattagatgaagagcaaccaatggagattgattaaacgaacatgccgcggaaccaattaaccaattctgattag
	27801rev-recSac-dORF7a	aggctaaaaagcacaaatagaagtcaattaatgaaagttcaatcaccgcggaggatgacgacgataagtaggg
d8-GLuc	27849fwd-recSac	cttgaactgcaagatcataatgaaacttgtcacgcctaaacgaaccgcggaaccaattaaccaattctgattag
	28493rev-recSac	gctgattttggggtccattatcagacattttagtttgttcgtttaccgcggaggatgacgacgataagtaggg
d8-GFP	d8-univFP-F	gaaacttgtcacgcctaaacgaacatggtgagcaagggcgaggag
	d8-univFP-R	ggtccattatcagacattttagtttgttcgtttacttgtacagctcgtccatgccg
	27849fwd-recSac	cttgaactgcaagatcataatgaaacttgtcacgcctaaacgaaccgcggaaccaattaaccaattctgattag
	28493rev-recSac	gctgattttggggtccattatcagacattttagtttgttcgtttaccgcggaggatgacgacgataagtaggg
d6-YFP	Yin-d6fwd	gcagtgacaatattgctttgcttgtacagtaagtgacaacagatggtgagcaagggcgaggagctg
	Yin-d6rev	gagtgttatcagtgccaagaaaagaataattttcatgttcgtttacttgtacagctcgtccatgccgag
after8-YFP	Yin-after8	gagtatcatgacgttcgtgttgttttagatttcatctaaacgaacatggtgagcaagggcgaggagctg

	29531-Nend-EYFP-KanRP	aacgtttatatagcccatctgccttgtgtggtctgcatgagtttacttgtacagctcgtccatgccgag
RT-qPCR	RdRp_fwd_nondeg	gtgaaatggtcatgtgtggcgg
	RdRp_rev_nondeg	caaatgttaaaaacactattagcata
	RdRp-probe VIC-BMN-Q535	caggtggaacctcatcaggagatgc
	sgGLuc-rev	cgatgttgaagtcttcgttgtt
	sgYFP-rev	agctcgaccaggatgggcac
	WHSA-00025F	ccaaccaactttcgatctcttgta
	sgYFP-probe 6-FAM/ZEN/3' IBFQ	accccggtgaacagctcctcgccct
	sgGLuc-probe 6-FAM/ZEN/3' IBFQ	tcggtgggcttggcctcggccaca

Oligonucleotides were synthesized by IDT, Biomers, or Thermoscientific. Long oligonucleotides (>50 nt) were purchased as Ultramer™ DNA Oligonucleotides (IDT).

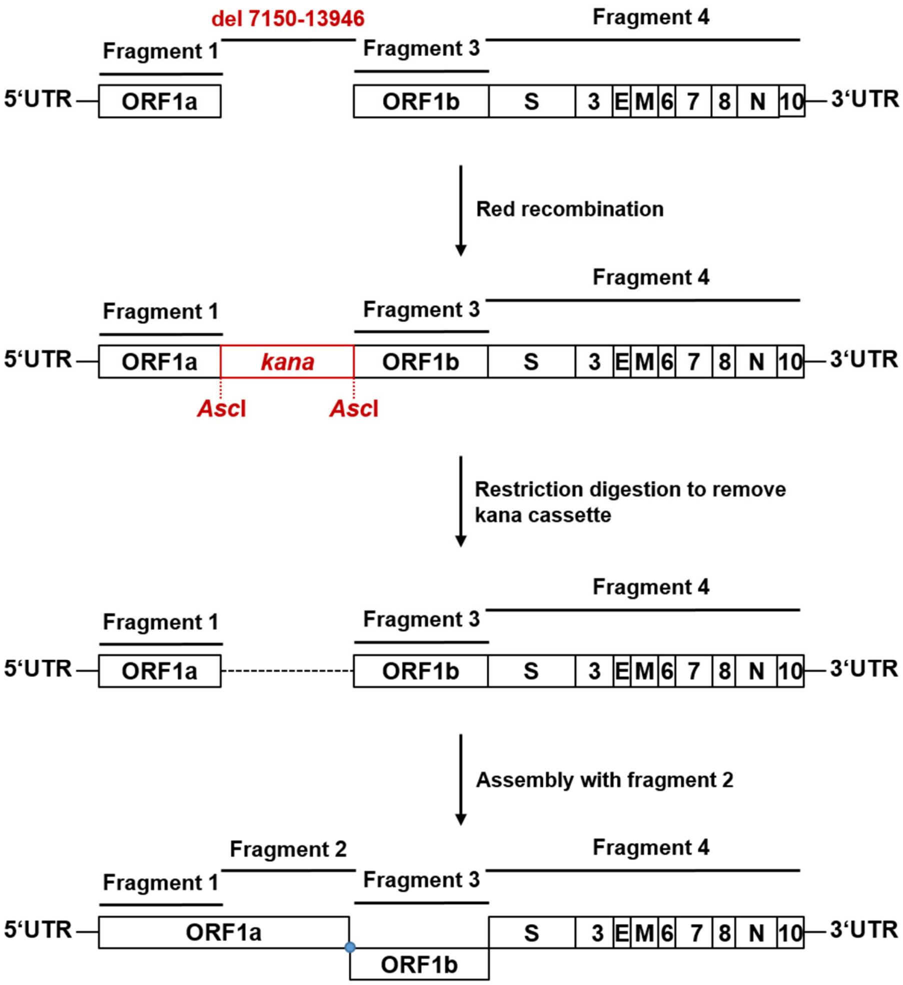

Figure S1. Schematic depiction of the complementation of pBSCo2 using Red recombination and assembly. A clone that lacked fragment 2 (deletion between nt 7150 to 13946), was used to generate the full-length bacmid pBSCoV2 BAC by Red recombination. A kanamycin (kana) resistance cassette with short sequence duplications to the site of deletion with flanking *Asc*I sites was inserted by recombination. After confirmation of correct clones, the kana cassette was removed by restriction digestion with *Asc*I and the missing fragment 2 was assembled with the digested linear backbone and transformed into *E.coli* GS1783, resulting in full-length pBSCoV2 clones.

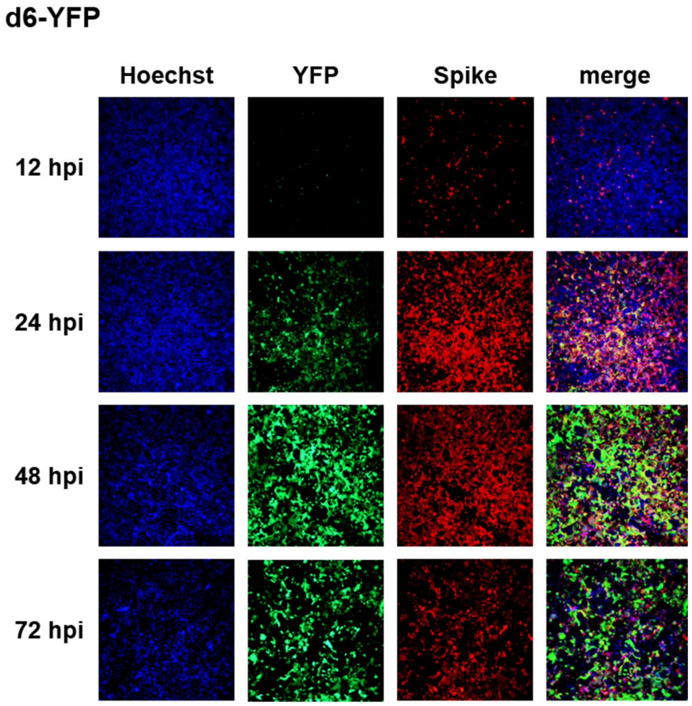

Figure S2. Visualization of d6-YFP reporter expression. CaCo-2 cells were infected with recSARS-CoV-2 d6-YFP (MOI = 0.005) and fixed at 12, 24, 48, and 72 hours post infection (hpi). Cells were immunostained with mAb against spike (red) and visualized for reporter expression (green). Hoechst 33342 was used for nuclear staining (blue). Imaging was performed with an ImmunoSpot Image Analyzer.

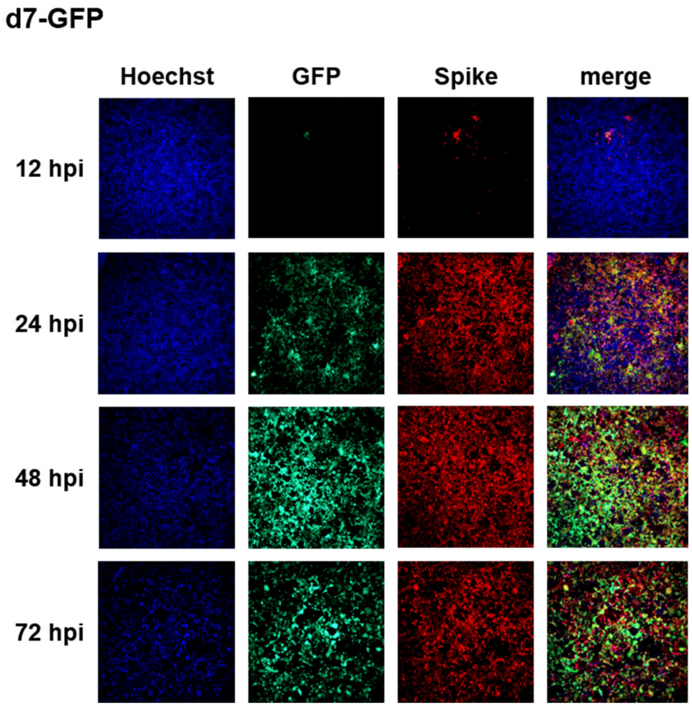

Figure S3. Visualization of d7-GFP reporter expression. CaCo-2 cells were infected with recSARS-CoV-2 d7-GFP (MOI = 0.005) and fixed at 12, 24, 48, and 72 hours post infection (hpi). Cells were immunostained with mAb against spike (red) and visualized for reporter expression (green). Hoechst 33342 was used for nuclear staining (blue). Imaging was performed with an ImmunoSpot Image Analyzer.

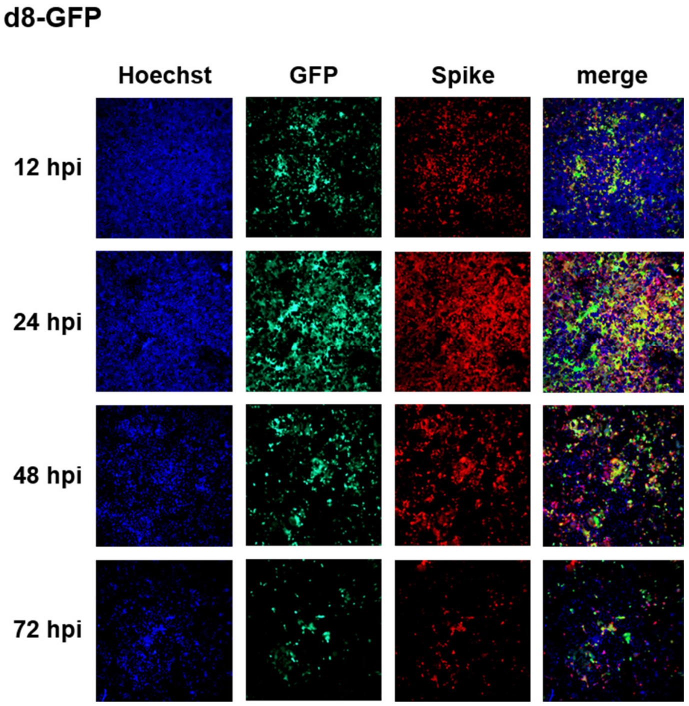

Figure S4. Visualization of d8-GFP reporter expression. CaCo-2 cells were infected with recSARS-CoV-2 d8-GFP (MOI = 0.005) and fixed at 12, 24, 48, and 72 hours post infection (hpi). Cells were immunostained with mAb against spike (red) and visualized for reporter expression (green). Hoechst 33342 was used for nuclear staining (blue). Imaging was performed with an ImmunoSpot Image Analyzer.

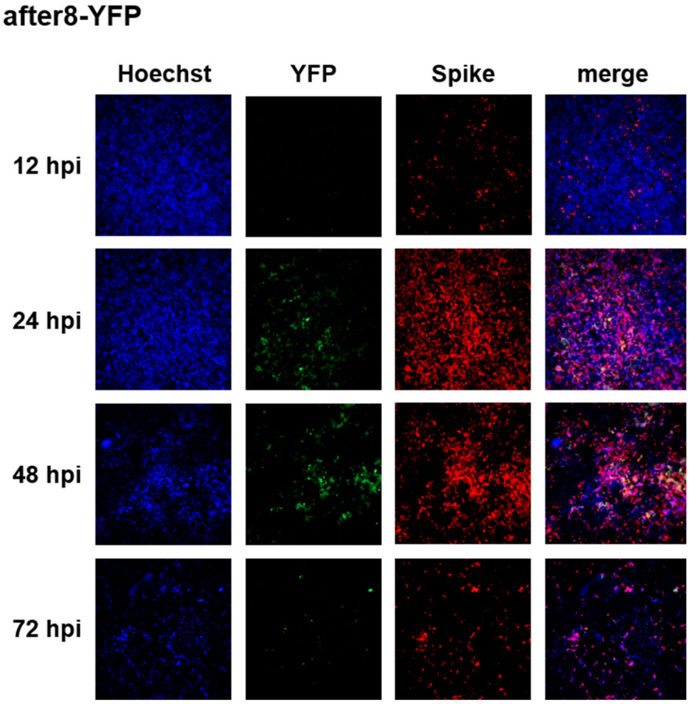

Figure S5. Visualization of after8-YFP reporter expression. CaCo-2 cells were infected with recSARS-CoV-2 after8-YFP (MOI = 0.005) and fixed at 12, 24, 48, and 72 hours post infection (hpi). Cells were immunostained with mAb against spike (red) and visualized for reporter expression (green). Hoechst 33342 was used for nuclear staining (blue). Imaging was performed with an ImmunoSpot Image Analyzer.

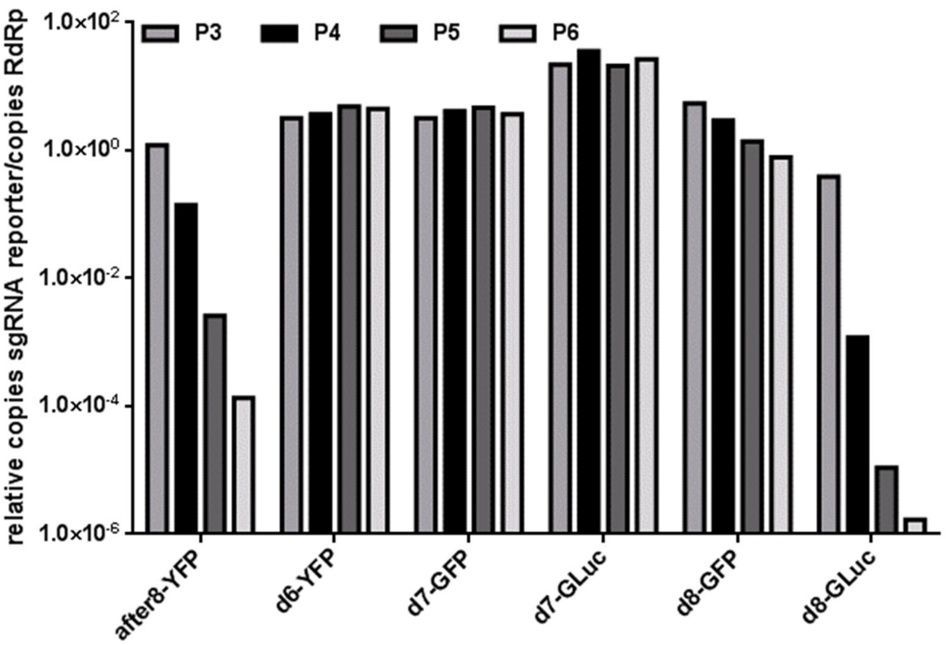

Figure S6. Reporter stability. CaCo-2 cells were infected with different reporter viruses. Passage (P) 2 virus stocks were further passaged on CaCo-2 cells for two days per passage. Total RNAs of infected cells were extracted from each passage. Presence of subgenomic reporter and genomic RNA (RdRp) was measured via RT-qPCR using specific probes. Ct values of reporter RNAs were normalized on viral load by RdRp Ct values. A representative experiment out of three independent experiments with similar results is shown.

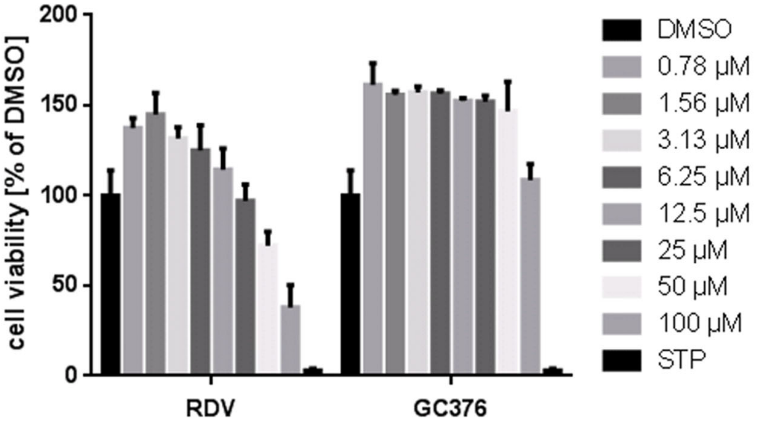

	RDV	GC376
CC50	72.7 ± 5.9 µM	> 100 µM

Figure S7. Cytotoxicity assay. Compound toxicities of Remdesivir (RDV) and GC376 were determined by Neutral Red Assay. CaCo-2 cells were incubated with the indicated concentrations for 3 days. Staurosporine (STP) at 10 µM served as control. The percentage of viable cells was calculated relative to DMSO-treated cells. Mean values of triplicates ± SD are shown.
